# Social Support and Diabetes Management Among Older American Indians

**DOI:** 10.1093/geroni/igab046.895

**Published:** 2021-12-17

**Authors:** R Turner Goins, Molly Grant, Kathleen Conte, Lisa Lefler

**Affiliations:** 1 Western Carolina University, Western Carolina University, North Carolina, United States; 2 Phoenix Counseling and Crisis Center, Gastonia, North Carolina, United States; 3 DePaul University, Chicago, Illinois, United States; 4 Western Carolina University, Cullowhee, North Carolina, United States

## Abstract

We examined social support among older American Indians in relation to their diabetes management. In-depth interviews were conducted with 28 participants aged ≥ 60 years who were members of a federally-recognized tribe. We examined professionally transcribed audio recordings with a systematic text analysis approach. Main sources of social support were family/friends, clinicians/formal services, community/culture, and spiritual/God. Most of the support was instrumental in nature, including food shopping, meal preparation, and medication management. Social support had both positive and negative influences diabetes management while there were some participants who lacked support. The four main social support types were present, including instrumental, emotional, informational, appraisal support. Value orientations among American Indian families command lateral-group relational behavior rather than autonomy and independence with extended social systems fostering interdependence. A deeper understanding is needed of how social relationships can be better leveraged to aid in the effective diabetes management among older American Indians.

